# Stress distribution patterns at mini-implant site during retraction and intrusion—a three-dimensional finite element study

**DOI:** 10.1186/s40510-016-0117-1

**Published:** 2016-01-18

**Authors:** Gautham Sivamurthy, Shantha Sundari

**Affiliations:** School of Dentistry, University of Dundee, Dundee, DD1 4HN Scotland; Department of Orthodontics, Saveetha Dental College and Hospital, Saveetha University, No. 162, Poonamallee High Road, Chennai, 600077 Tamil Nadu India

## Abstract

**Background:**

The purpose of this study was to evaluate the stress patterns produced in mini-implant and alveolar bone, for various implant dimensions, under different directions of simulated orthodontic force, using a three-dimensional finite element method.

**Methods:**

Eight finite element (FE) models of mini-implant and bone were generated with insertion angles of 30° and 60°, diameters of 1 and 1.3 mm, and lengths of 6 and 8 mm. A simulated constant orthodontic force of 2 N was applied to each of these FE models in three directions simulating anterior retraction, anterior intrusion and retraction, and molar intrusion.

**Results:**

Comparison of the maximum von Mises stress in the mini-implant showed that the 1-mm diameter produced significantly high stress, and the amount of stress produced was more for a mini-implant inserted at an angle of 60°. The cortical bone showed that high stresses were generated for the 1-mm-diameter mini-implant and on increasing the insertion angulation from 30° to 60°, the stress produced increased as well. The comparison of von Mises stress in the cancellous bone was insignificant as the amount of stress transmitted was very low.

**Conclusions:**

The 1-mm-diameter mini-implants are not safe to be used clinically for orthodontic anchorage. The 1.3 × 6 mm dimension mini-implants are recommended for use during anterior segment retraction and during simultaneous intrusion and retraction, and the 1.3 × 8 mm dimension mini-implant is recommended for use during molar intrusion. All mini-implants should be inserted at a 30° angle into the bone for reduced stress and improved stability.

## Background

In the past three decades, the finite element (FE) method has become an increasingly useful tool for the prediction of stress effect on the implant and its surrounding bone, especially in the field of implant dentistry, and with more accurate computer simulation and modeling technologies, it has interested dental researchers even further. The FE method is way of getting a numerical solution to a specific problem. It involves cutting a structure into several smaller pieces to describe the behavior of each element in a simplified way and then reconnecting them at nodal points. Using associative functions like stress and deformation, the mechanical behavior of these elements can be numerically studied [1, 2].

Mini-implants have become an essential armamentarium component in resistance to unwanted tooth movement during orthodontic treatment. While providing absolute anchorage, these devices are used for specific periods of time and rely only on mechanical retention with the surrounding bone. Thus, it is imperative that mini-implants remain stable during their period of use to provide sufficient anchorage during treatment.

Mini-implant failures have been reported as an issue primarily related to infection and secondarily to biomechanical parameters such as length, diameter, and the angle at which the mini-implants are inserted into the bone [3–5]. By understanding the stresses produced along the surfaces of a mini-implant and in the surrounding bone, the design and placement of the mini-implant can be optimized and therefore help minimize failures within the mouth.

Previous numerical and in vitro studies have evaluated the dimensional parameters but have not combined all biomechanical factors to investigate the most suitable dimensions and insertion angle for better success [6–9]. The application of force, the amount of force applied, and the direction of force all have significant effects on the amounts of bone produced around mini-implants [10].

Therefore, the objective of this study was to analyze the stress distribution patterns which developed in and around a mini-implant on application of a simulated constant orthodontic load of 2 N [11, 12] and to determine the most suitable combinations of length, diameter, and insertion angle of the mini-implant for use during various simulated tooth movements and also experimented using thinner diameter mini-implants to check suitability for use.

## Methods

The present study involves the consideration of four primary elements in the development of the three-dimensional finite element model: (1) mini-implant design—which includes the length, diameter, and pitch of the screw; (2) geometry of the mini-implant and bone structures—the geometry and design of the mini-implant head and taper and the thickness of cortical and cancellous bones to be modeled; (3) establishment of three-dimensional finite element model of the mini-implant—i.e., FE model of the mini-implant inserted in bone; and (4) material properties—Poisson’s ratio and Young’s modulus for the mini-implant (titanium) and cortical and cancellous bones. For this study, the material properties were derived from related research [8] (Table 1).

**Table 1:** Material properties used construction the models

Material	Young’s modulus (MPa)	Poisson’s ratio (*v*)
Titanium	110,000	0.35
Cortical bone	15,000	0.3
Cancellous bone	1,500	0.3

**Fig. 1:**
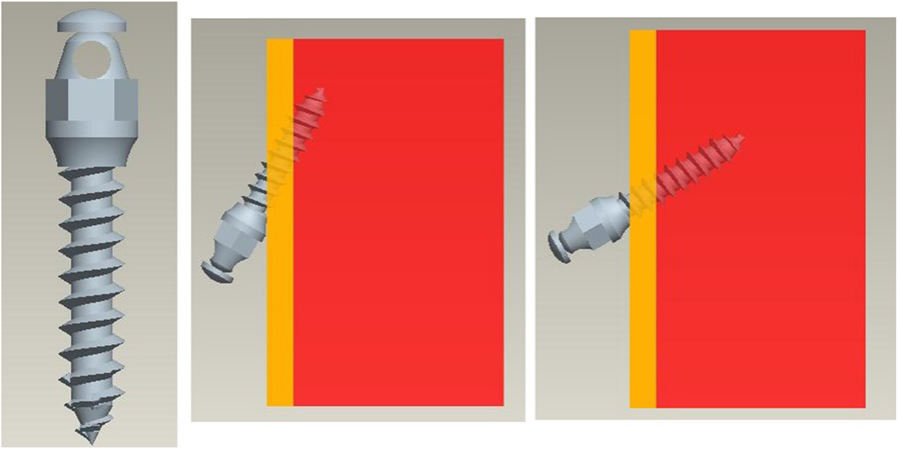
Three-dimensional solid model of the mini-implant and bone: three-dimensional solid model of a 35-mm section of the alveolar bone of the posterior maxilla with a single self-drilling titanium mini-implant with a small head-type and tapered screw. Cortical layer of the bone depicted in *orange* and the cancellous bone in *red*

The geometric morphology of the mini-implants was designed according to the dimensions and measurements obtained from AbsoAnchor (Dentos Inc., Korea). We designed the mini-implant as a small head-type tapered pure titanium screw with external diameters of 1 and 1.3 mm, lengths of 6 and 8 mm, threaded deepness flight depth of 0.2 mm, threaded angle of 60°, and thread interval of 0.5 mm, with angulation of insertion to the vertical plane, at 30° and 60° (Fig. 1). Four FE models of the mini-implants with the abovementioned combinations were designed.

A three-dimensional solid model of a 35-mm section of the alveolar bone of the posterior maxilla with a single self-drilling titanium mini-implant (Fig. 1) and subsequent models with varying lengths, diameters, and implant angulation were created. We used the ANSYS Workbench (version 11.0) finite element analysis program to generate the solid model, create the mesh of individual elements, and perform the post-processing to calculate the stresses and strains.

Gap elements were defined between the mini-implant and at all peripheral nodes of the bone with zero coefficient of friction which afforded no movement in all directions [1]. Diameters of the implant thread and the hole were made identical. Bone elements were arbitrarily designed to be a block 8 × 14 × 10 mm in dimension to be large enough to assess the stresses and strains surrounding the mini-implant.

**Fig. 2:**
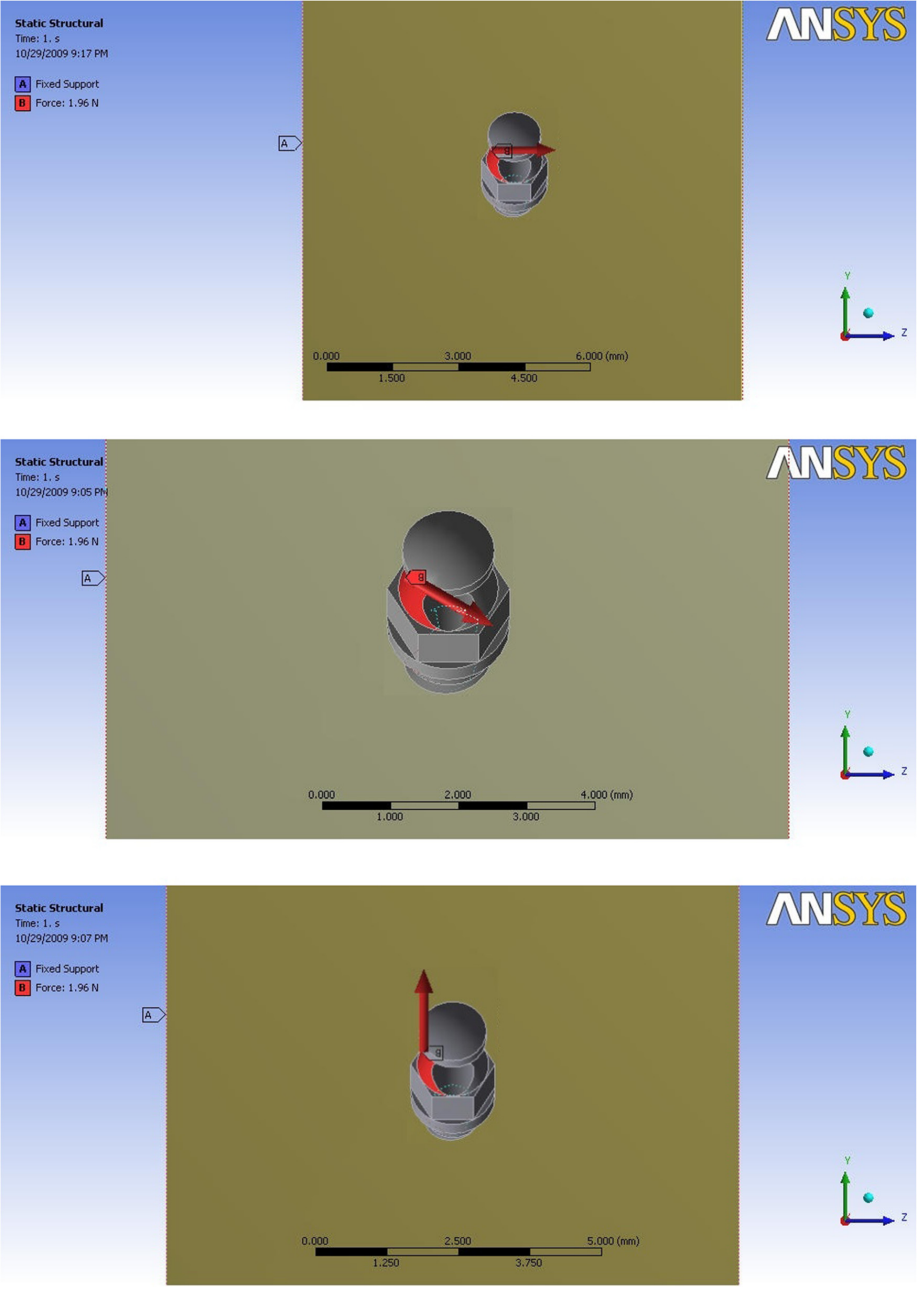
Direction of orthodontic force to head of mini-implant: force applied at 90° to vertical plane (simulating anterior retraction), 30° to vertical plane (simulating anterior intrusion and retraction), and 90° to horizontal plane (simulating molar intrusion)

The ANSYS software was used to mesh the mini-implant and bone models and to perform the finite element analysis on the mini-implants with insertion angles of 30° and 60°, diameters of 1 and 1.3 mm, and lengths of 6 and 8 mm, therefore generating eight FE models and grouping as listed in Table 2. A simulated constant orthodontic force of 2 N was applied to each of these FE models and the stress distribution on the implant-bone interface was analyzed, assuming that the force is applied to the head of the mini-implant. The direction of applied orthodontic force was simulated to clinical situations of anterior retraction (by applying a force at 90° to the vertical plane of the mini-implant), anterior intrusion and retraction (30° to the vertical plane of the mini-implant), and molar intrusion (90° to the horizontal plane of the mini-implant) (Fig. 2).

**Table 2:** List of FE models and groups

Group	Description
1a	Mini-implant model 1.3 × 6 mm at 30° insertion
1b	Mini-implant model 1.3 × 6 mm at 60° insertion
2a	Mini-implant model 1.3 × 8 mm at 30° insertion
2b	Mini-implant model 1.3 × 8 mm at 60° insertion
3a	Mini-implant model 1 × 6 mm at 30° insertion
3b	Mini-implant model 1 × 6 mm at 60° insertion
4a	Mini-implant model 1 × 8 mm at 30° insertion
4b	Mini-implant model 1 × 8 mm at 60° insertion

## Results

The stress distribution for mini-implants in this study was evaluated according to the von Mises stress hypothesis, in MPa units (megapascal). A color scale served to evaluate quantitatively the stress distribution in the bone, i.e., cortical and cancellous bones, and the mini-implant. The stress scale runs from blue to red, where blue depicts no stress (0 MPa) and red indicates the area of highest stress.

**Fig. 3:**
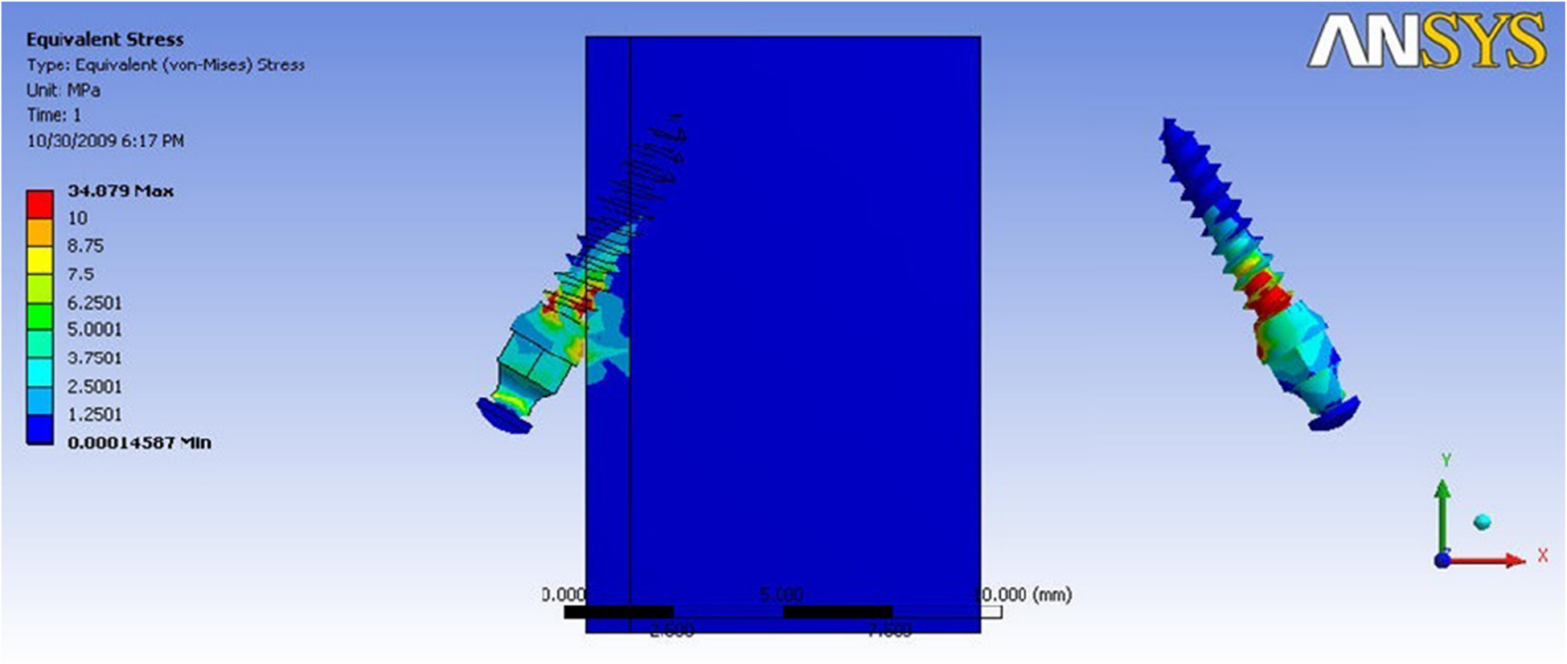
von Mises stress distribution for mini-implant group 1a—anterior retraction: von Mises stress distribution seen at the bone-implant interface and in the mini-implant alone. Maximum stress seen in the mini-implant was at 34.82 MPa

**Fig. 4:**
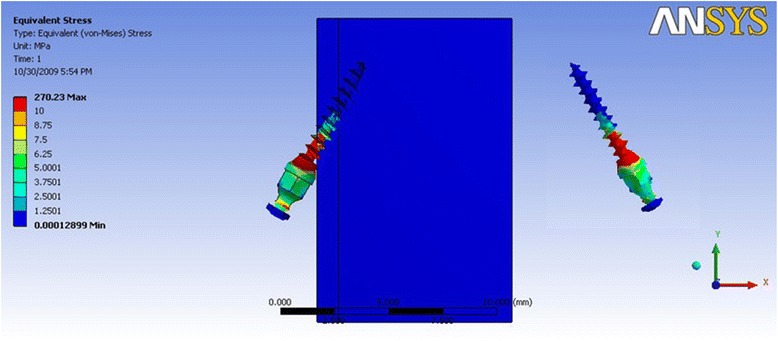
von Mises stress distribution for mini-implant group 3a—anterior retraction: von Mises stress distribution seen at the bone-implant interface and in the mini-implant alone. Maximum stress seen in the mini-implant was at 270.12 MPa

In Table 3 where the direction of force simulated anterior segment retraction, it is evident that the distribution of stress was concentrated in the neck of the mini-implant and the cortical bone was subjected to higher stresses as compared to the cancellous bone. The stress values are higher when the mini-implant is inserted at a 60° angle as compared to a 30° insertion angle. The cortical bone was shown to be stressed least in mini-implant model 1a (Fig. 3), whereas mini-implant model 3a showed the highest stress value (Fig. 4). Also, groups 3 and 4 showed stress highly concentrated at the neck of the mini- implant, in the contact between the thread and cortical bone.

**Table 3:** Maximum von Mises stress (MPa) for force simulating anterior retraction

Dimension of mini-implant	Maximum stress in mini-implant	Maximum stress in the cortical bone	Maximum stress in the cancellous bone
Group 1a (Fig. 3)	34.82	22.66	0.24
Group 1b	41.005	32.23	0.31
Group 2a	39.82	26.63	0.27
Group 2b	38.97	28.02	0.30
Group 3a (Fig. 4)	270.12	106.36	0.35
Group 3b	143.84	54.63	0.31
Group 4a	209.4	84.67	0.32
Group 4b	213.2	78.23	0.41

**Fig. 5:**
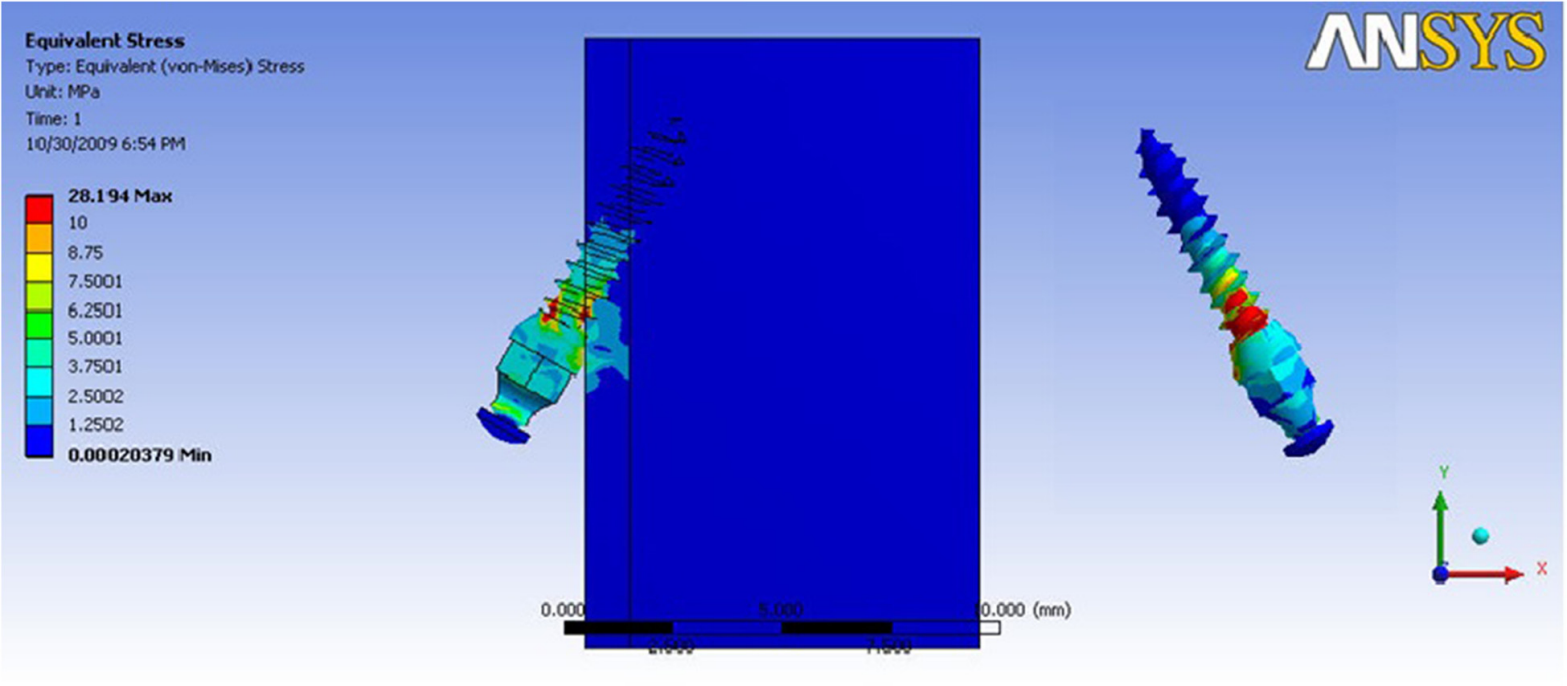
von Mises stress distribution for mini-implant Group 1a—anterior intrusion and retraction: von Mises stress distribution seen at the bone-implant interface and in the mini-implant alone. Maximum stress seen in the mini-implant was at 28.0 MPa

**Fig. 6:**
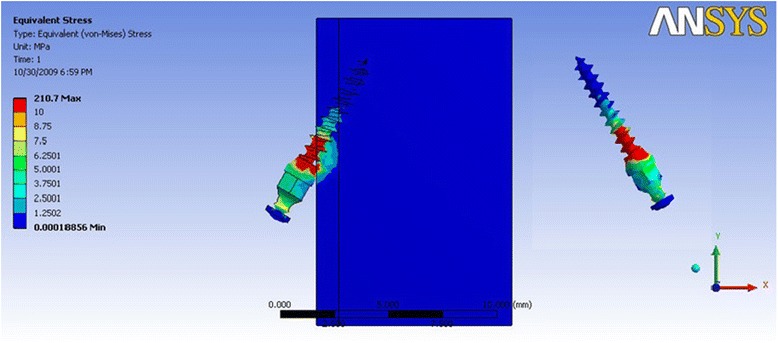
von Mises stress distribution for mini-implant group 3a—anterior intrusion and retraction: von Mises stress distribution seen at the bone-implant interface and in the mini-implant alone. Maximum stress seen in the mini-implant was at 210.22 MPa

In Table 4 where the direction of force simulated anterior segment intrusion and retraction, the model 1a (Fig. 5) showed the least amount of stress whereas mini-implant model 3a showed the highest stress values both in the mini-implant and cortical bone (Fig. 6). Overall, for mini-implants with a dimension of 1 mm, the stress distribution was relatively much higher, as compared to the 1.3-mm diameter.

**Table 4:** Maximum von Mises stress (MPa) for force simulating anterior intrusion and retraction force

Dimension of mini-implant	Maximum stress in mini-implant	Maximum stress in the cortical bone	Maximum stress in the cancellous bone
Group 1a (Fig. 5)	28.0	17.22	0.22
Group 1b	43.34	29.33	0.31
Group 2a	36.24	18.84	0.24
Group 2b	36.46	23.04	0.34
Group 3a (Fig. 6)	210.22	79.74	0.066
Group 3b	125.06	62.29	0.54
Group 4a	159.6	73.58	0.32
Group 4b	156.26	77.09	0.44

**Fig. 7:**
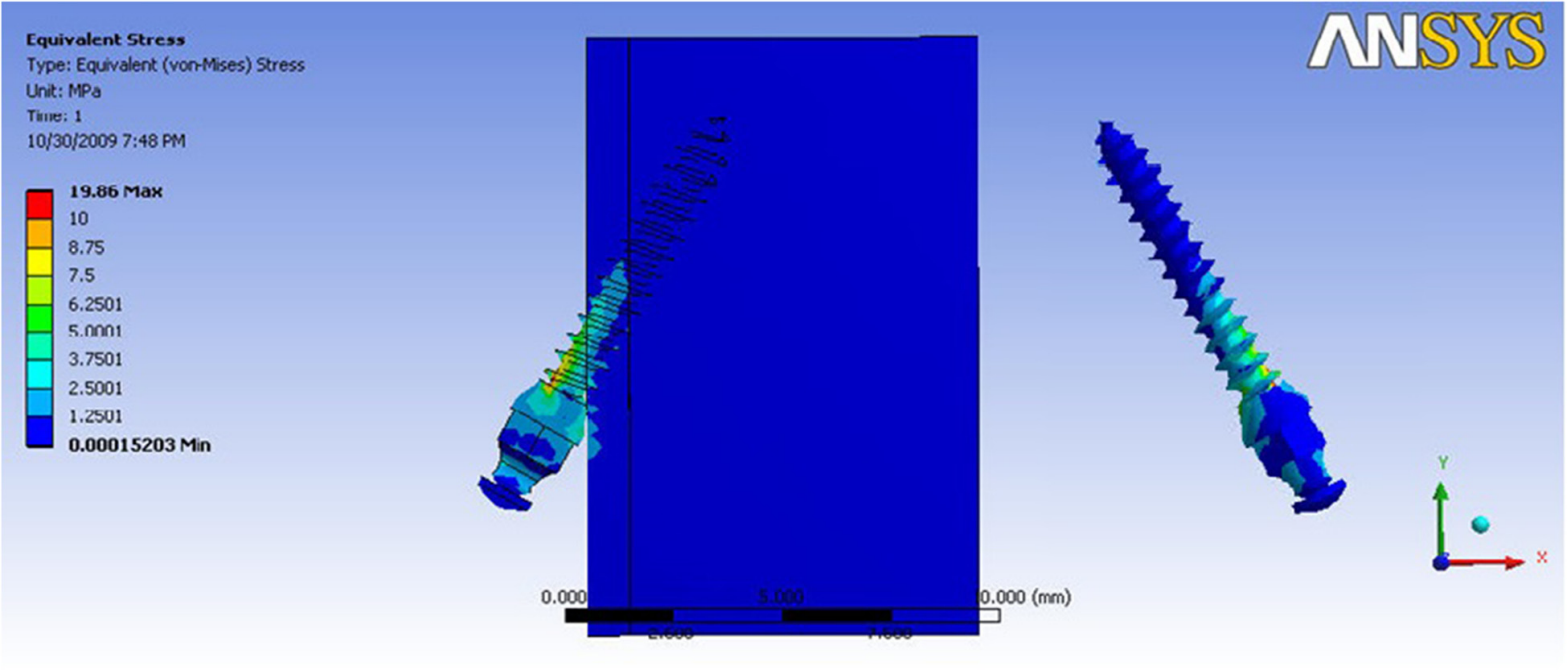
von Mises stress distribution for mini-implant group 2a—molar intrusion: von Mises stress distribution seen at the bone-implant interface and in the mini-implant alone. Maximum stress seen in the mini-implant was at 19.85 MPa

**Fig. 8:**
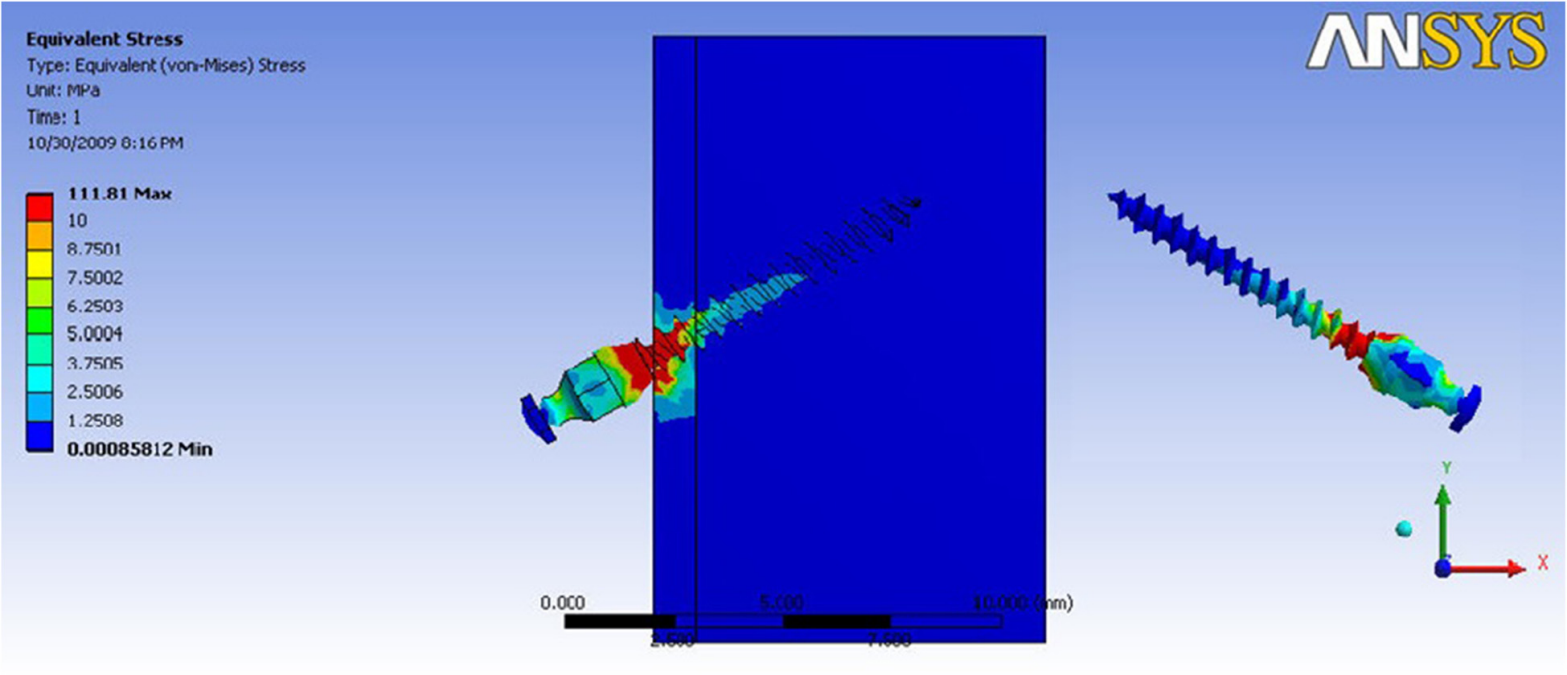
von Mises stress distribution for mini-implant group 4b—molar intrusion: von Mises stress distribution seen at the bone-implant interface and in the mini-implant alone. Maximum stress seen in the mini-implant was at 111.27 MPa

In Table 5 where the direction of force simulated molar intrusion and retraction, mini-implant model 2a (Fig. 7) showed a stress distribution which was relatively less as compared to the other dimensions of mini-implants. The amount of stress concentrated in the cortical bone was seen to be least in mini-implant 2a, whereas model 4b (Fig. 8) showed the highest stress value, which was for the 1-mm-diameter mini-implant, with stresses concentrated around the neck of the mini-implant. Model 3a showed a lesser stress value around the neck, among the 1-mm-diameter group of mini-implants.

**Table 5:** Maximum von Mises stress (MPa) for direction of force simulating molar intrusion

Dimension of mini-implant	Maximum stress in mini-implant	Maximum stress in the cortical bone	Maximum stress in the cancellous bone
Group 1a	21.09	17.85	0.22
Group 1b	29.34	28.92	0.44
Group 2a (Fig. 7)	19.85	14.15	0.22
Group 2b	37.16	24.83	0.40
Group 3a	81.19	75.46	0.12
Group 3b	98.2	51.26	0.70
Group 4a	75.17	47.25	0.14
Group 4b (Fig. 8)	111.27	89.89	0.59

### Surface area calculation

The above table depicts the amount of surface area taken up in the cortical bone and in the whole bone, by the mini-implant models used in this study. According to the FE model results, the mini-implant model 2b had the highest surface area of the bone covering it (29.45 mm^2^). The least amount of whole bone covering the mini-implant was seen for model 3a, which was at 12.24 mm^2^. It is important to understand that from an orthodontic point of view, mini-implant anchorage is mainly derived from the cortical bone.

Therefore, when the amount of surface area of the cortical bone alone surrounds the mini-implants, it is evident that the mini-implant model 1a showed a much higher area of 7.76 mm^2^ which was taken up, as compared to the other models.

## Discussion

Various kinds of mini-implants have been used for orthodontic anchorage reinforcement ever since Kanomi et al. [13] suggested titanium mini-implants as intraoral anchorage devices. Wu et al*.* [14] studied the success rate of mini-implants, concluded that careful diameter selection for different locations is essential, and recommended an implant diameter equal to or less than 1.4 mm in the maxilla, and diameter larger than 1.4 mm in the mandible was suggested for better orthodontic anchorage. An assortment of geometric designs based on length, diameter, composition of alloy, thread pitch, taper, and shapes of head are available and are being tried clinically, and usually, the insertion angle of mini-implants varies most often according to the clinician’s preference. Therefore, it is necessary to compare the efficacy in terms of stress induced in the metal and bone among the mini-implants of various geometric designs and insertion angles, when they are subjected to force application and directions, according to the clinical requisite (e.g., retraction force, intrusion and retraction, extrusive force).

The finite element method is an effective tool to identify optimal design parameters and allow for improved mini-implant designs. The comparative analysis of numerical and experimental data of orthodontic mini-implants by Chatzigianni et al*.* [15] revealed a tendency that the finite element analysis offers a promising alternative to experimental procedures. Hence, this study aimed to evaluate stress distribution pattern among varying mini-implant dimensions of length, diameter, and insertion angulation, when subjected to orthodontic loads directed to simulate clinical situations of anterior segment retraction, anterior intrusion and retraction, and molar intrusion in a mathematical model using the FE method.

To simulate orthodontic force levels, a force of 2 N was used in this numerical analysis since previous studies used a load application of 2 N; but the study by Chatzigianni et al*.* [12] showed that differences in the results can also be explained by the applied force level and a difference was found between the mini-implant groups in their study when a high force of 2.5 N was applied. Further analysis of their data revealed that the level of 1 N could be defined as the threshold for differentiation; but even they agree that with the majority of clinical studies cited, load application was 2 N or less and therefore no clear discrimination between force levels could be observed.

It has long been recognized that both the implant and bone should be stressed within a certain range for physiological homeostasis. This mechanical stress in turn causes strain in the bone tissue which is defined as a relative change in length, whether lengthening or shortening. The degree of the strain correlates with stress and the bone’s mechanical characteristics. According to Frost [16] (2003), the amount of strain can be divided into various ranges, permitting us to predict the effects on the bone. The lower limit of the bone’s equilibrium (i.e., of the load range within which, due to continuous bone remodeling processes, as much bone tissue is formed as is resorbed) is roughly 50–100 μStrain (1-2 MPa). Below this limit, (due to underuse), the result is bone resorption. The upper limit of this range is roughly 1000–1500 μStrain (20 MPa). Bone formation is the initial response above this limit. Additional strain, however, leads to micro-fissures and micro-fractures in the bone tissue, which, at roughly 3000 μStrain (60 MPa), surpasses ongoing repair processes leading to bone resorption. Therefore, if the mini-implant displacement exceeds the specified physiologic limit, it is likely to cause a micro-fracture of the bone trabecula and result in absorption, and necrosis of the osseous tissue in implant-bone interface ultimately leads to the failure of the mini-implant.

### Stress analysis on mini-implant metal

In our study, stress values observed on the mini-implant have shown that for dimensions 1.3 × 6 mm and 1.3 × 8 mm, insertion angles at 30° and 60° had a minimum value of 19.85 MPa (Table 5) and a maximum value of 43.34 MPa (Table 4), which were well within the acceptable fatigue limit of titanium of 193 MPa [17]. FEM studies by Zhang et al. [18] have shown similar results with 30° insertion angulation of mini-implants producing a decreased stress value of 22 MPa. They also concluded that when the mini-implant was embedded with a tilted angle of 30°, the length would be doubled correspondingly to penetrate the cortical bone. Therefore, while the tilted angle is decreased, the contact area of the micro-implant and cortical bone is increased to enhance the stability of micro-implants accordingly.

The stress values on mini-implant dimensions 1 × 6 mm and 1 × 8 mm of 30° insertion angulation and 1 × 8 mm of 60° insertion angulation, however, showed a higher range *above* the acceptable fatigue limit (210–270 MPa) (Tables 3 and 4). The other parameters (3b of Table 3, 3b, 4a and 4b of Table 4) showed a higher range but *within* acceptable fatigue limits of titanium (125–159 MPa). However, Table 4 depicting molar intrusion simulation did show a *lower range* between 75 and 111 MPa which was also within acceptable fatigue limits of titanium. Miyawaki et al. [4] (2003) reported a higher success rate for mini-implants of diameters 1.2 and 1.3 mm, than for the 1.6-mm diameter. He also reported 0 % success rate when 1-mm-diameter mini-implants were used, stating a reason of higher chance of fracture when used and therefore advocated that it was not suitable for clinical use. It was found in studies by Melo Pithon et al. [9] that the torsional strength values increased as their diameters also increased. However, such a reduced size also decreases the mechanical strength, thus reducing the maximum torsional strength and resulting in deformation and fracture.

According to Lemieux et al. [19], during mini-implant length selection, the clinician should consider the important trade-off between anchorage and risk of placement complications or damage to the tissues. Longer mini-implants enable more anchorage; however, they are associated with a higher risk of damage to neighboring structures. Placement depth and bone density at the site of mini-implant placement are the best predictors of primary stability.

### Stress analysis on the cortical bone

The stress distribution patterns in the cortical bone showed that, on inserting the mini-implant of dimension 1.3 mm (inclusive of 6- or 8-mm length) at a 30° angulation, the stress distribution in the cortical bone was only marginally decreased, as compared to the 60° insertion angulation. The minimum stress distribution values obtained in the cortical bone for 30° insertion angulation were 22.66 MPa (Table 3), 17.22 MPa (Table 4), and 14.15 MPa (Table 5), for the three directions of force application studied. These values were in accordance with results obtained from studies by Motoyoshi et al. [8]*.* The highest stress values obtained were for the 60° insertion angulation—32.23 MPa (Table 3), 29.33 MPa (Table 4), and 28.92 MPa (Table 5), in all three directions of force application. However, it is pertinent to note that the difference in the minimal and maximal values was only marginal and well within Frost’s [16] mechanostat values.

For the 1-mm-diameter mini-implant (Tables 3, 4, and 5), however, the stress values observed in the cortical bone for both 30° and 60° insertion angles ranged between 47.25 and 89.89 MPa, except for group 3a of Table 3, which showed a maximum value of 106.36 MPa, which was also within Frost’s mechanostat values.

Kyung et al. [20] advocate mini-implant insertion at 30°–40° to increase the surface contact between the implant and bone and allow the insertion of a longer screw in the available bone depth. Also, Deguchi et al. [21] believed that angling the implant at approximately 30° would increase contact with as much as 1.5 times more to the cortical bone. Pickard et al. [22] studied the effect of mini-implant orientation on stability, and they found that the more closely the long axis of the mini-implant approximates the line of applied force, the greater the stability of the implant and the greater its resistance to failure.

The effect of diameter on mini-implant stability has been compared by many authors. Miyawaki et al. [4] (2003) and Seon et al. [7] (2003) reported that the diameter of the mini-implant affected the success rate the most, as compared to the other dimensional parameters. The diameter also affects the placement and removal of the mini-implant, which in turn affects the stability as well. Barros et al. [23] showed that an increase in mini-implant diameters significantly influenced the increases of placement torque and fracture torque on quantities that progressively reduced the fracture risk. Lee et al. [24] in their study showed that mini-implants with larger diameters and tapered shapes caused greater microdamage to the cortical bone. This they believe in turn might affect bone remodeling and the stability of the mini-implants. Lui et al. [25] believe that the screw diameter was the dominant factor for mini-implant mechanical responses. They showed both that bone stress and screw displacement decreased with increasing screw diameter and cortex thickness and decreasing exposed length of the screw, force magnitude, and oblique loading direction. Differences in implant diameter could also influence other aspects of implant integration, such as induction of remodeling, and could interact with other factors of mini-implants (e.g., when the implant is loaded) to influence microdamage [26].

Melsen [27] believes that the length of a mini-implant should be determined by depth and quality of the bone, screw angulation, transmucosal thickness, and adjacent vital structures. Short screws in regions with thick soft tissues, such as the palatal mucosa, can easily become dislodged and therefore these authors advocate use of lengths greater than 6 mm. Baek et al. [3] advocate the use of longer mini-implants in areas of thicker cortical bone, for increased primary stability. Seon et al. [7] (2003) reported that the maintenance of the mini-implant is more reliable on the length and since the cortical surfaces of the maxillary buccal area are thinner and less compact than those of the mandible and therefore require longer mini-implants. The study by Motoyoshi et al. [28] showed that screws of 1.2-mm diameter and at least 8-mm length are preferable, because they are stable and minimize the risk of root damage; and Upadhaya et al. [29] have shown that when using a mini-implant with a length of 8 mm for molar intrusion, vertical dimension control is maintained.

### Stress analysis on the cancellous bone

Stress distribution in the cancellous bone when analyzed between Tables 3, 4, and 5 showed values ranging between 0.06 and 0.59 MPa, which could be considered as least stress induced in the cancellous bone during simulated orthodontic tooth movement. Studies by Zang et al. [18] have shown similar results where stress values in the cancellous bone ranged between 0.63 and 0.56 MPa. Based on their findings, they concluded that the cortical bone would receive larger stress while forces were conducted from micro-implant to the implant-bone interface owing to the higher elastic modulus of the cortical bone compared with that of the spongy bone.

The stress patterns obtained from Table 3, 4, and 5 showed that the values in the cortical bone and cancellous bone were well within the normal limit for all dimensions of mini-implants considered in the present study but not in the metal. The high values of stress perceived in the metal particularly of 1-mm mini-implant maybe unfavorable for orthodontic use. This could be implying a possibility for a fracture at the neck during orthodontic loading and hence not recommended for clinical use. Also, results from Jiang et al.’s [30] study showed that the increases of the diameter and length reduced the maximum equivalent stresses in cortical and cancellous bones and mini-implant.

### Surface area of mini-implant-bone interface

The surface area was calculated for the amount of alveolar bone surrounding the various dimensions of mini-implants used in this study. (Table 6). This calculation was done in relation to two aspects of the bone surrounding the mini-implant, i.e., the surface area of cortical bone alone around the mini-implant and the surface area of whole bone (cortical and cancellous bones) around the mini-implant.

**Table 6:** Comparison of Surface area (mm^2^) of cortical bone and whole bone surrounding the models

FE model	Surface area (mm^2^)	
	Cortical bone	Whole bone
1a	7.76	14.2
1b	4.89	19.75
2a	6.84	23.98
2b	5.12	29.45
3a	3.88	12.24
3b	3.23	15.22
4a	3.84	19.05
4b	3.13	21.91

On considering the whole bone-implant surface area, Table 6 revealed that 1.3 × 8 mm at both insertion angles had the greatest implant-bone interface surface area of 29.45 and 23.98 mm^2^, respectively. Kanomi [13] however, believed that, from an orthodontic point of view, when mini-implants are used for skeletal anchorage, it is the cortical bone which provides this. Also, Muhsin et al. [31] (2011) believe that to obtain a balanced intrusion, root surface area should be considered when determining the appropriate forces. Therefore, it is important to take into account the surface area of the cortical bone surrounding the mini-implant rather than the whole bone. Also, Lin et al. [32] have shown that the exposure length of the mini-implants significantly influenced bone stress; increased exposure lengths resulted in greater bone stresses adjacent to the mini-implant.

On considering the cortical bone-implant surface area, it was evident that the surface area increased when the mini-implant was inserted at a 30° angulation only, rather than when it was used at a 60° angulation in each combination of 1- and 1.3-mm mini-implants, (more so in the 1.3-mm combination than in the 1-mm combination of mini-implants). Between 1.3- and 1-mm mini-implants, the mini-implant dimension of 1.3 mm (inclusive of 6- and 8-mm length) at a 30° insertion angulation showed the highest surface area of the cortical bone at 7.76 and 6.84 mm^2^, respectively. The other mini-implant dimensions, i.e., 1.3 mm at 60° insertion angulation and all combinations of 1-mm-diameter mini-implants at both 30° and 60° insertion angulations, ranged between 3 and 5 mm^2^ only (Table 6).

## Conclusions

Within the limitations of this study, the following conclusions were drawn:

1. The comparison of the maximum von Mises stress in the mini-implant showed that the 1-mm diameter produced significantly high stress and the amount of stress produced was more for a mini-implant inserted at an angle of 60°, with the stress being concentrated at the neck and head of the mini-implant, immaterial of length 6 mm or 8 mm.
2. The comparison of stresses in the cortical bone showed that high stresses were generated for the 1-mm-diameter mini-implant, and on increasing the insertion angulation from 30° to 60°, the stress produced increased as well, with the stress being concentrated in the cortical bone around the threads of the mini-implant.
3. The comparison of von Mises stress in the cancellous bone was insignificant as the amount of stress transmitted was very low. The comparison of von Mises stress for 6-mm length of mini-implant was decreased when the direction of force simulated anterior segment retraction and anterior segment intrusion and retraction, whereas the 8-mm-length mini-implant produced stress which was comparatively lower in clinical situations of molar intrusion.
4. The 1.3 × 6 mm dimension mini-implants are recommended for use during anterior segment retraction and during simultaneous intrusion and retraction, and the 1.3 × 8 mm dimension mini-implant is recommended for use during molar intrusion. All the mini-implants should be inserted at a 30° angle into the bone for reduced stress and improved stability.
5. From this study, we noted that even though all 1-mm mini-implant models underwent greater stress as compared to the 1.3-mm models, most stress values were still within the acceptable fatigue limit of titanium. The study has limitations since we have not considered all biomechanical parameters which could affect stability of the mini-implant, for example, torque during insertion and removal of the mini-implant, which may induce additional stress and fatigue of the metal; but from observations of the stress values in the cortical bone, the 1-mm mini-implant produces significantly greater values and hence we conclude that 1-mm-diameter mini-implants are not safe to be used clinically for orthodontic anchorage, until further research proves otherwise.
